# Future Life-Cycle Greenhouse Gas Emission Scenarios for the Austrian Building Stock: A Systematic Approach

**DOI:** 10.1021/acs.est.4c12138

**Published:** 2025-03-07

**Authors:** Nicolas Alaux, Bernhard Steubing, Guillaume Habert, Marcella Ruschi Mendes Saade, Alexander Passer

**Affiliations:** † Working Group Sustainable Construction, Institute of Structural Design, Graz University of Technology, 8010 Graz, Austria; ‡ Institute of Environmental Sciences (CML), Leiden University, 2300 RA Leiden, Netherlands; § Chair of Sustainable Construction, Institute of Construction and Infrastructure Management, ETH Zurich, 8093 Zurich, Switzerland

**Keywords:** scenario development, carbon reduction strategy, prospective life-cycle assessment (pLCA), net-zero emissions, sufficiency measure, building stock modeling

## Abstract

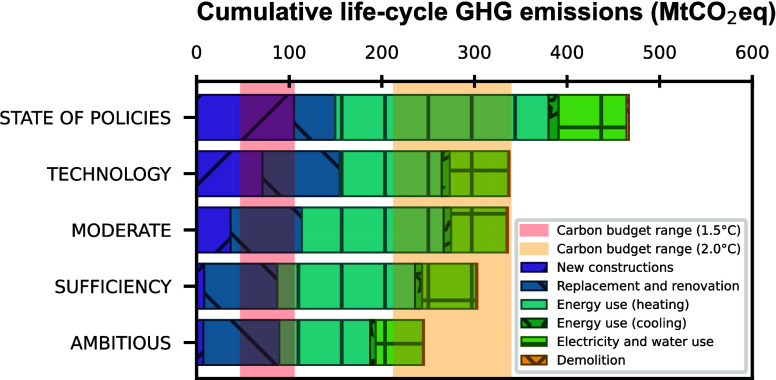

Building stock modeling can be used to identify trajectories that do not exceed the remaining carbon budget and support science-based pathways. A systematic approach is used from the field of prospective life-cycle assessment, which is based on systems thinking, to develop scenarios for the Austrian building stock that consider life-cycle greenhouse gas emissions. The influential parameters of the model are identified; their interactions are classified; quantitative future assumptions are adopted; and five scenario narratives are created. A maximum emission reduction of 90% from 2023 to 2050 is revealed. In comparison, leaving current policies in place would lead to a trajectory that reduces emissions by only 66%. Three additional scenarios achieve emission reductions between 84 and 86% by 2050, which may be compatible with the 2 °C carbon budget using an equal-per-capita approach. These scenarios represent different societal choices based on ambitious sufficiency (e.g., behavioral change), technological measures (e.g., a change in the industry), or both, with less effort from all actors. To ensure that Austria contributes to staying within the remaining carbon budget, policy makers are urged to systematically and quickly incorporate sufficiency into their policies and enable the necessary investments in carbon dioxide removal technologies.

## Abstract 2

Most building stock scenarios do not assess life-cycle emissions and lack clear scenario development methods. In this study, a systematic approach is used to construct future life-cycle emission scenarios for the Austrian building stock.

## 1 Introduction

The existence of man-made climate change is indisputable. The reports of the Intergovernmental Panel on Climate Change (IPCC) emphasize the clear influence of human activities on climate change.^1^ Recent data show that human-induced global warming is increasing at a pace that is unprecedented in the instrumental record, with an average observed warming of 1.19 °C in 2023.^2^ A profound, rapid and sustained reduction in greenhouse gas (GHG) emissions (especially CO_2_) is necessary.^1^ To address this challenge, in 2015, 195 countries agreed to limit global warming to 1.5–2 °C above preindustrial levels by 2050 (known as the Paris Agreement). The nearly linear relationship between cumulative anthropogenic CO_2_ emissions and subsequent temperature increases makes it possible to determine global CO_2_ budgets, i.e., the amount of CO_2_ that can be emitted before 2050 while still meeting the +1.5 °C (or 2 °C) target.^3^ Once the budget is exhausted, CO_2_ emissions must fall to net zero.^1^ As these budgets consist of cumulative CO_2_ emissions, they can be translated into different CO_2_ emission pathways.^4^

Buildings play a key role in this process. The construction and operation of buildings is responsible for 21% of global GHG emissions.^5^ Building-related emissions are cross-sectoral in nature and are divided into operational emissions, which include emissions that originate from the daily functioning of buildings, and embodied emissions, which are usually emitted at a specific time during the life cycle of a building. To assess the potential for mitigating building-related emissions correctly, it is essential to collect more accurate information on building stock.^6^ This is why an increasing number of environmental building stock models that simulate interactions between embodied and operational GHG emissions has been developed.^7,8^ In Austria, however, these emissions are still generally assessed separately,^9^ with existing bottom-up models focusing on either energy consumption in buildings^10^ or material stocks and flows.^11^ This lack of a common scope can be detrimental to the development of GHG emission reduction trajectories.^12^

Building stock modeling can indeed be used to define trajectories that stay within the carbon budget limits. This is done on the basis of future scenarios, which are helpful for collecting multiple plausible visions of the future.^13,14^ While many studies have focused on energy system models^15^ or material flow analysis (MFA) of building stocks,^16^ more recent approaches have focused on linking these models to prospective life-cycle assessment (LCA).^17^ However, this linkage remains mostly technical, multiplying the material and energy flows by time-dependent characterization factors without the use of systematic methods to develop scenarios.^7,8^ Scenarios are an important aspect of the field of prospective LCA, where frameworks have been created for scenario development in small- or medium-scale prospective LCA studies.^18,19^ These methods need to be integrated into building stock modeling to ensure the quality of the results. In this work, this gap is addressed through the use of a systematic approach from the field of prospective LCA, which is based on systems thinking, to develop scenarios for the Austrian building stock considering life-cycle GHG emissions.

## 2 Methods

The methodology applied follows the SIMPL methodology proposed for prospective LCA studies.^19^ The rationale for this choice relates to its development by a transdisciplinary team, which is essential for providing a comprehensive understanding of a system as cross-sectoral as the building stock. Additionally, it has been tested extensively by numerous LCA practitioners and revised on the basis of the feedback received, which ensures a certain level of robustness for the method. The structure of the methodology is as follows: (i) The goal and scope of the analysis are described in section 2.1. (ii) The inventory analysis is described in section 2.2. (iii) The impact assessment and concept of the remaining carbon budget are explained in section 2.3. (iv) The scenario development process is explained in section 2.4, including key factor identification, key factor analysis, and scenario generation.^19^ (v) The uncertainties of the model are explored via sensitivity analyses, as explained in section 2.5.

### 2.1 Goal and Scope Definitions

The aim of this study is to address the following research questions:

(1) What are the expected GHG emission reductions of the implemented climate policy targets on the Austrian building stock?
(2) Which mitigation strategies are required for the Austrian building stock to limit global warming to approximately 1.5–2.0 °C?

While the first question is rather predictive in nature (“How will the future develop”), the second question is normative (“How should the future develop”).^20^ The scenario definition perspective that is adopted is therefore of a hybrid nature. The scope includes the GHG emissions of all buildings (residential and non-residential) that are physically located in Austria (860 million m^2^ net floor area in 2023) from 2023 to 2050. Both the operational emissions (energy consumption for heating, cooling, electricity and water use in the building) and the embodied emissions (production of materials, transport, construction, replacement, renovation, demolition and waste processing) are considered. These emissions are fully accounted for in the year in which the activity in the building stock takes place (e.g., if a building is renovated in 2040, it is assumed that all embodied emissions associated with the renovation are emitted in 2040). The cumulative GHG emissions, i.e., the sum of all annual GHG emissions stemming from the Austrian building stock from 2023 to 2050, are used as a functional unit for comparison with the remaining carbon budget.

### 2.2 Inventory Analysis

**Figure 1:**
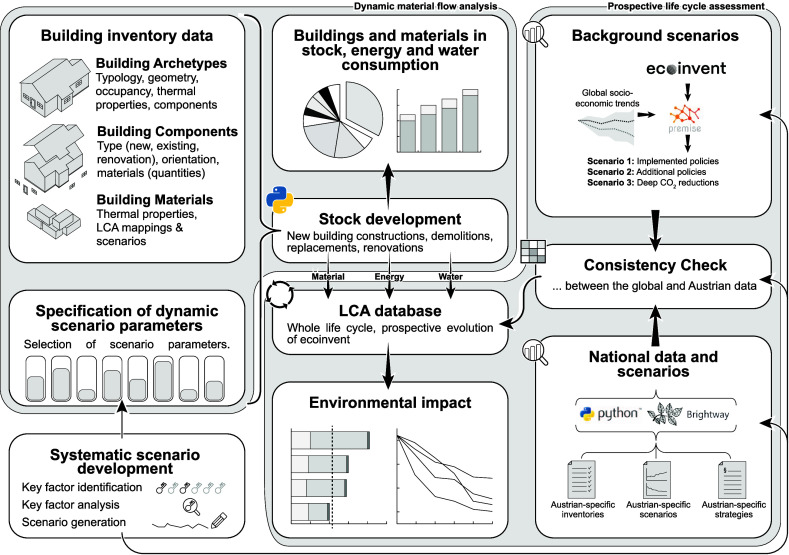
Modeling framework used in this study, which combines dynamic MFA, prospective LCA and systematic scenario development to project the environmental impacts of the Austrian building stock.

The modeling framework for the inventory and impact assessment is shown in Figure 1. Here, a short overview of the Austrian building stock model that is used for this study, PULSE-AT v2.0, is provided; more detailed information is available in a previous publication.^21^ The input data of the model are structured in the following way: (i) Buildings are grouped into building archetypes, which are defined on the basis of the period in which they were built (e.g., 1981–1990), building typology (e.g., single-family house), main structural material (e.g., concrete) and energy performance level (e.g., standard). Each archetype is then assigned specific information, such as energy consumption, geometry and heating system data. (ii) For material inventorization, each archetype is divided into its components (e.g., exterior walls) and materials (e.g., plaster and concrete), which is typical for LCAs of buildings.^22^ Information on building components and materials are gathered from building catalogues^23−26^ and expert consultations. (iii) These input data are then imported into a Python-based model that simulates the activities of the building stock (new buildings, renovations, replacements, energy consumption, and demolitions) on the basis of the specification of dynamic scenario parameters. Using dynamic MFA, the material, energy and water flows stemming from these building stock activities are calculated on an annual basis. These input data are validated on the basis of statistical data and scientific literature, as explained in a previous publication.^21^

These flows are then multiplied by the characterization factors from the LCA database to calculate the annual environmental impact of the building stock. The LCA database is created as follows: (i) The set of assumptions provided by the premise package v2.2.4^27^ are used to consistently change electricity generation, goods transport and heat supply across the entire ecoinvent database v3.9.1 cutoff^28^ according to the global scenarios of the integrated assessment model (IAM) of the REgional Model of Investment and Development (REMIND). All transformations available in this premise version were applied. Four scenarios for the REMIND SSP2 series are used: the NPi scenario, which accounts for the implemented national policies; the NDC scenario, which accounts for the national commitments following the Paris Agreement; the PkBudg1150, which results in a 66% chance of limiting global warming to 2 °C above preindustrial levels; and the PkBudg500, which results in a 66% chance of limiting global warming to 1.5 °C above preindustrial levels. (ii) To better simulate Austrian conditions, the markets for electricity (low, medium and high) are overwritten with the latest scenarios of the Austrian Environment Agency,^9^ and inventories for the district heating mix are added (following projections from the same source), as are those for the future production of cement, bricks and steel.^29−31^ This is performed via Brightway2^32^ as an LCA framework and a custom Python script. The inventories are provided in a scenario difference file,^33^ some of which could be made available upon reasonable request.

### 2.3 Impact Assessment and the Remaining Carbon Budget

The remaining global carbon budget represents the total global amount of CO_2_ that can still be emitted to remain within a specific global warming limit with a certain probability.^3^ The challenge usually resides in translating this global carbon budget into budgets that are specific to buildings or building stocks. This was addressed in a previous publication,^34^ which provided a carbon budget for the life cycle emissions of the Austrian building stock; this carbon budget can be translated into a GHG budget, assuming that 5% of the GHG emissions of buildings are non-CO_2_ emissions.^5^ The carbon budget can be given as a range, depending on the likelihood of reaching the temperature target and the allocation mechanism used to derive it. According to a calculation based on the equal-per-capita approach, the carbon budget ranges from 49 to 104 and 214 to 338 Mt CO_2_eq for temperature targets of 1.5 and 2.0 °C, respectively. This approach was chosen because it is reported as a decent compromise that seems to be widely accepted by the community to operationalize the derivation of the carbon budget in the short term.^35^ The term “carbon budget” will be used consistently in this paper, although it now actually refers to a “GHG budget” in MtCO_2_eq.

Once the budget is exhausted, in 2050, CO_2_ emissions must fall to zero net.^1^ This means that CO_2_ removal technologies must be implemented to ensure that the remaining emissions in 2050 are offset. The assessment of CO_2_ removal technologies is beyond the scope of this paper, but is addressed in the discussion. GHG emissions are therefore reported with the climate change fossil impact indicator, using the environmental footprint (EF) method v3.1 EN15804.^36^ This means that our approach represents a conservative view of the concept of the remaining carbon budget.

### 2.4 Scenario Development

#### 2.4.1 Key Factor Identification

The identification of the influential parameters of the system was performed in two steps: first, the technological parameters that were included in the model were identified and, second, the surrounding parameters that influence the technological parameters were identified. A preliminary list of technological parameters was compiled on the basis of existing knowledge, literature reviews^37^ and studies of building stock models.^7,8,38^ To facilitate the creation of scenarios, each of these parameter groups was subdivided into more precise parameters and assigned to the main actor who has the most influence on the parameter (architects, industry, owners, or tenants). Although further addressed in section 2.4.2 and fully provided in the Supporting Information, the ranges of values for these parameters in 2050 are also provided below for transparency. The parameters are as follows:

- Space demand reduction (owners and tenants): Average living area per capita (41–55.3 m^2^/cap), share of new single-family houses (5–40%), and decreased demand for commercial buildings (office and trade) (8–22%).
- Vacancy reduction (owners, tenants): Use of empty, secondary dwellings (residential buildings), or building units (non-residential buildings) (0–35%).
- Renovation rate (owners): Thermal renovation rate (light, medium, or deep package) (1.4–2.5%), non-thermal renovation rate (8–15%), frequency of heating system replacement (15–25 years), share of heating systems upgraded with a lower GHG-emitting system (30–100%).
- Energy demand reduction (architects, owners, and tenants): Decrease in personal heating consumption due to behavioral change (0–20%), share of new buildings with advanced energy efficiency standards (5–100%).
- Renewable energy supply (industry): Share of renewable electricity in the production mix (92–100%), share of renewable energy (including waste) in district heat production (76–100%).
- Design and material optimization (architects): Share of material use optimized in designs (concrete and steel) (5–30%), share of new buildings without basements (35–85%), share of new timber buildings (22–40%), and reduction in plastic-based insulation (20–40%).
- Material production improvement (industry): Share of fuel consumption switched to alternative fuels (including electrification and hydrogen) (30–100%), share of process emissions mitigated via carbon capture and storage (cement, brick, and steel production) (0–90%), share of clinker in total cement used (41–60%), and share of scrap steel in steel production (30–75%).
- Transport and construction improvement (industry): Reduction in transportation distances (0–37%), share of vehicles with energy efficiency improvements (8–22%), share of alternative fuels in construction machinery fuel used (28–99%), and share of construction machines optimized (6–12%).
- Waste prevention (architects, owners, and industry): Share of repurposed buildings (buildings that were about to be demolished that are instead renovated) (30–90%), reduction in construction waste on site (0–60%).
- Waste recovery (architects and industry): Recycling rate (wood, gypsum, and insulation) (30–100%) and reuse rate (concrete and steel) (5–29%).

The surrounding parameters (or societal context) are important for the contextualization of the system and support the scenario description (the narratives); however, they are not necessarily included in the model. First, a preliminary list of surrounding parameters was identified through a review of the literature on the global sensitivity analysis of building stock modeling and scenario development.^10,39^ Then, an internal workshop was organized with transdisciplinary experts from Graz University of Technology to refine the list of parameters. As suggested by the SIMPL methodology,^19^ the PESTEL checklist was used to incorporate political, economic, sociological, technical, environmental and legal factors. The seven identified most important surrounding parameters are as follows:

- Legislation and policies (including subsidies, taxes, bans, coherence, motivation, and willingness, both at the national and supranational levels).
- Population growth (due to e.g., fertility and migration).
- Cultural inertia (traditions, habits, unwillingness to change, and scepticism).
- Fairness of the transition (minimum decent quality and living standards, income distribution, diversity, poverty, and cost of the transition).
- Climate change effects (increased temperatures, extreme events, environmental degradation, and biodiversity loss).
- Awareness and education (communication, attractiveness, and climate anxiety).
- Economic and international situation (gross domestic product, trade, resource availability, global cooperation, and wars and armed conflicts).

**Figure 2:**
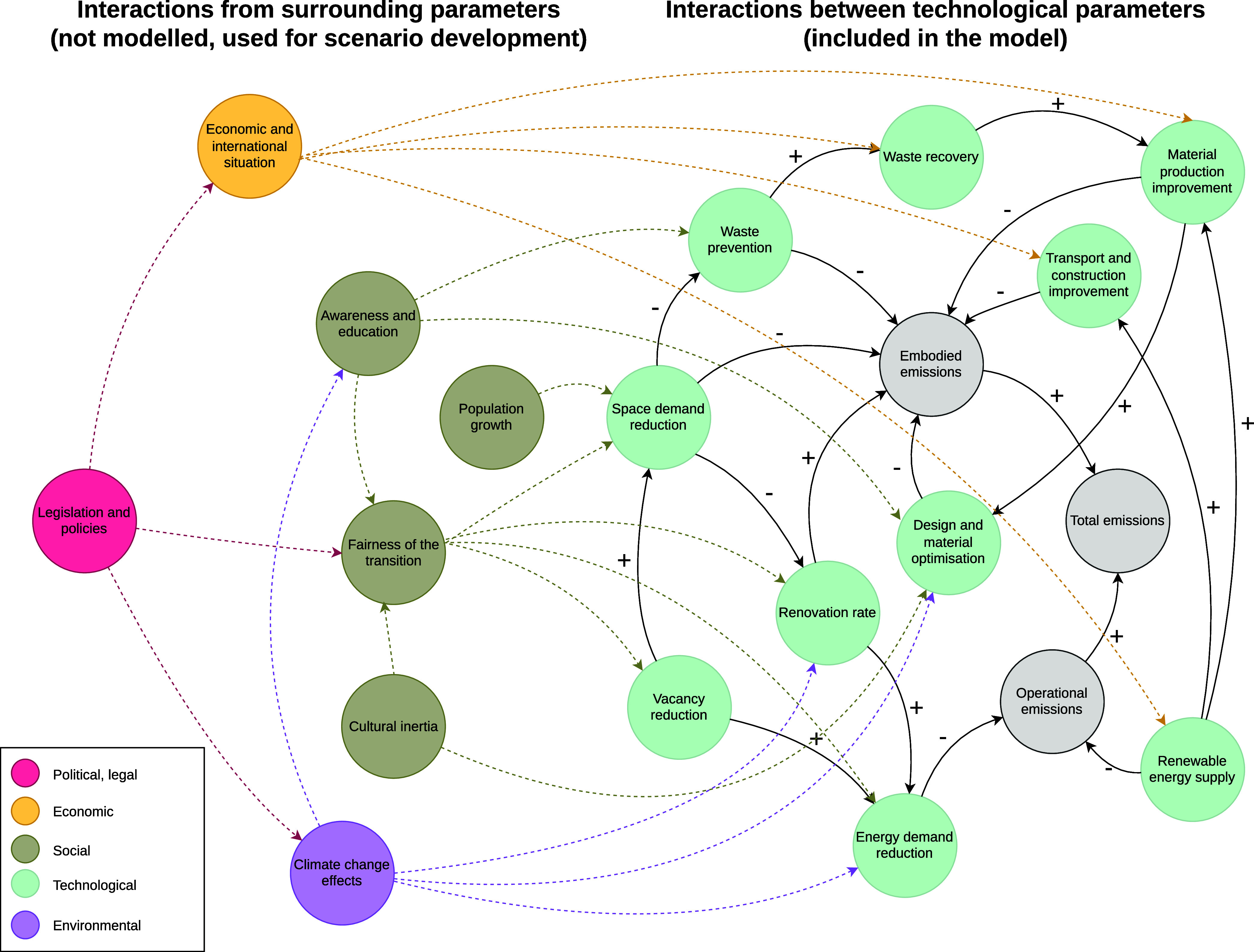
Causal loop diagram showing the expected interaction of the surrounding parameters with the technological parameters of the model. The technological parameters are shown in the green circles. The interactions between the technological parameters are fully included in the model. The surrounding parameters are color-coded according to their PESTEL categories. Their interactions with the technological parameters are theoretical and not included in the model, but they are used to inform the development of scenario narratives.

The expected interaction of these surrounding parameters with the technological parameter groups is shown in the simplified causal loop diagram (CLD) in Figure 2.

#### 2.4.2 Key Factor Analysis

The next step was to collect quantified future assumptions for each relevant technological parameter of the model. For each parameter, the future assumptions for 2030, 2040, and 2050 were quantified. A linear development over the individual decades was assumed. Owing to the uncertainty behind the development of these parameters, the future assumptions for each decade were quantified at three levels of ambition: low, medium, and high. Therefore, each parameter had nine possible future values. The future assumptions were created by combining different sources, favoring Austrian-specific data when available. Examples of Austrian-specific data include those from national statistics and projections,^40,41^ reports from national institutions,^9,42^ and national research projects.^30,31,43^ European literature was the second choice for data.^37,44,45^ Finally, in cases where no data were found, assumptions were made. The full list of parameter groups, parameters, future assumptions and sources can be found in the Supporting Information.

The implementation of these parameters depends on whether they interact with the life cycle inventory (LCI) database. Space demand reduction, vacancy reduction, renovation rate, energy demand reduction, design and material optimization, waste prevention, and waste recovery do not require modifications of the LCI database. Instead, they define the reference flows to satisfy the functional unit. These parameters are provided as inputs of the building stock model and are implemented in the model (“specification of dynamic scenario parameters” in Figure 1). The implementation of a renewable energy supply, material production improvement, and transport and construction improvement require that modifications are made to the LCI database. This is performed according to the procedure defined in section 2.2 (“complemented with national data” in Figure 1).

#### 2.4.3 Scenario Generation

Scenarios are typically based on narratives, which describe the principal driving forces within it.^20^ The narratives are based on the created causal loop diagram and are inspired by research on earth system modeling^46^ as well as French transition scenarios.^47^ When national scenarios are combined with scenarios from the IAMs, ensuring consistency between the scenario narratives is important. Therefore, qualitative consistency checks were performed to determine which scenarios could be combined while a consistent narrative was maintained. The five scenarios that were investigated to answer the research questions are as follows:

(1) State of policies (POL): This scenario is designed to capture the impact of the implemented policies, which is a challenge due to the lack of quantified targets in the policy documents.^48^ We rely on the Austrian Environment Agency’s “With existing measures” scenario, which considers policy measures from January 2022.^9^ From this report, we can estimate the renovation rate, the energy carriers for space heating and the share of renewable energy in electricity and district heating. The other assumptions underlying this scenario are not fully available to the public, which is why we assume that all actors involved have low ambition. The surrounding parameters are assumed to follow previous trends. The REMIND SSP2-NPi background scenario was used. As current policies might not be enough to reach the GHG emission reduction targets necessary for climate change mitigation,^9^ this scenario is expected to lead to radiative forcing of 4.5 W/m^2^ by 2100 (RCP4.5). Therefore, to account for climate change effects, a decrease of 7% in the heating demand corresponding to buildings was applied, as well as an increase of 61% in the corresponding cooling demand by 2050 (following the evolution of heating and cooling degree days,^49^ as a simplification).
(2) Technology (TECH): Society relies on technological progress to overcome environmental challenges without significantly changing lifestyles. Owing to strong cultural inertia, there are few space demand reduction, energy demand reduction, and vacancy reduction efforts. Few behavioural changes are observed among owners and tenants. Legislation and policies are pushing industry to rapidly reduce emissions to ambitious levels from 2030 onwards, which will strongly affect material production improvement, waste recovery, transport and construction improvement, and the renewable energy supply. Owing to limitations on the renewable energy supply, the renovation rate of buildings is also a major point on the political agenda. Owing to this strong mitigation from industry, awareness and education among architects is increasing, leading to changes in design and material optimization and waste prevention, but only at a moderate level of ambition due to remaining cultural inertia. The REMIND SSP2-PkBudg1150 background scenario was used (RCP2.6), and accordingly, a decrease of 6% in the heating demand corresponding to buildings by 2050 was applied, as was an increase of 50% in the cooling demand by 2050.^49^
(3) Sufficiency (SUF): Significant societal changes lead to increased sufficiency. Behavioural changes among tenants and owners are quickly enabled by a combination of appropriate legislation and policies that ensure the fairness of the transition, as well as increased awareness and education. High levels of ambition are seen for vacancy reduction, space demand reduction, energy demand reduction, and waste prevention. Owing to the high level of energy demand reduction, there is less pressure on renovation rates, for which a moderate level of ambition is observed. The REMIND SSP2-NDC background scenario was used, and similarly, a decrease of 6% in the heating demand corresponding to buildings by 2050 was applied, as was a 50% increase in the cooling demand by 2050.^49^
(4) Moderate (MOD): Society is moving towards a mix of efficiency and sufficiency. Technological developments take time, whereas behavioural changes can be implemented in the short term. This is reflected in legislation and policies as well as increases in awareness and education. Cultural inertia is easier to overcome if all actors are involved and willing to ensure fairness in the transition. Owing to the early involvement of all actors, the level of ambition can remain in the moderate range, facilitating the implementation of the transition. The REMIND SSP2-NDC background scenario is used, and a decrease of 6% in the heating demand corresponding to buildings by 2050, as well as an increase of 50% in the cooling demand by 2050,^49^ is applied.
(5) Ambitious (AMB): This is an extreme scenario in which all actors maximise their mitigation efforts in each decade to pursue the maximum achievable mitigation potential. The REMIND SSP2-PkBudg500 background scenario is used. Owing to the strong ambition present in this scenario, negligible effects of climate change on building heating and cooling demands are expected.

**Figure 3:**
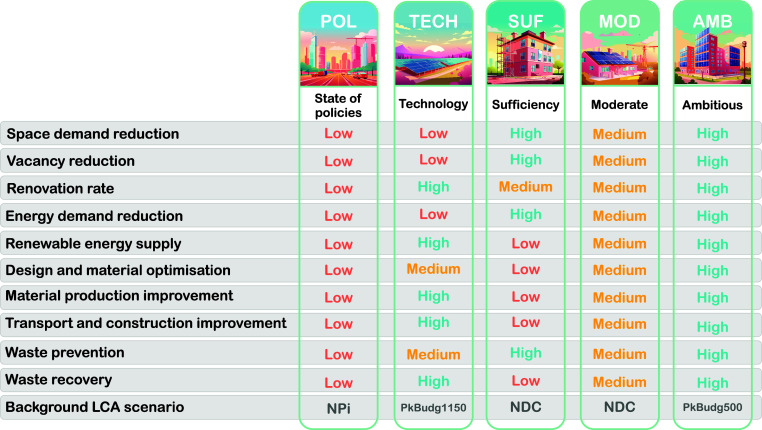
Summary of the five scenario narratives. The low, medium and high ambition levels are linked to the quantitative numbers provided in the Supporting Information. The state of policies is taken as a reference, which is why all ambition levels are low. This should not be seen as a judgment of the ambition level corresponding to current policies. The pictures used to visualize the scenarios were generated via Adobe Sensei GenAI.

In all the scenarios, population growth always follows the main statistical projection,^41^ which is consistent with the SSP2 narrative used as the LCA background scenario. Future assumptions for all parameters for each year from 2023 to 2050 can be found in the Supporting Information. Figure 3 shows a summary of the five scenarios.

### 2.5 Sensitivity Analysis

Various sensitivity analyses were performed to further explore the uncertainties behind the scenario modeling results. First, for the ten technological parameters shown in Figure 3, the elementary effect method of Morris,^50^ which was accessed via the SAlib package,^51^ was used to identify which parameters have the greatest impact on GHG emission reduction. Following previous work, the number of trajectories was set to ten and the number of levels to four.^52^ Uncertainty ranges were created for 2050 on the basis of the low and high values of each parameter, which were assumed to be uniformly distributed; and a linear evolution from 2023 to 2050 was chosen. To investigate the influence of the background LCA scenario, the REMIND SSP1-PkBudg500, REMIND SSP5-PkBudg500, and IMAGE SSP2-RCP19 scenarios were additionally applied to the AMB scenario. Finally, in section 4.3, the influence of the use of different allocation principles for the carbon budget calculation is discussed.

## 3 Results

### 3.1 Yearly GHG Emissions for the Scenarios

**Figure 4:**
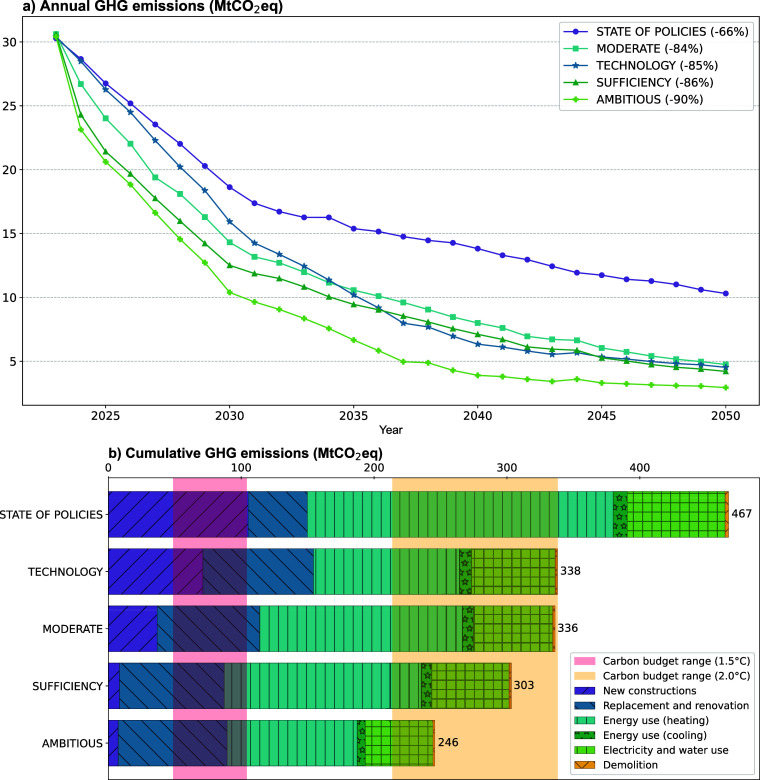
(a) Annual GHG emissions for the investigated scenarios and (b) cumulative GHG emissions for the scenarios from 2023 to 2050 compared with the remaining carbon budgets for different temperature increases.

The annual GHG emissions for the scenarios analyzed are shown in Figure 4a for each year from 2023 to 2050. The GHG emission reduction that is achieved by 2050 compared with that in 2023 is shown next to the name of each scenario. In the POL scenario, a 66% reduction in GHG emissions is achieved, which is significantly lower than those of the other four scenarios. In the TECH, SUF, and MOD scenarios, reductions of 84–86% are achieved. The AMB scenario shows the maximum potential reduction in GHG emissions that can be achieved, namely, 90%. In terms of temporal dynamics, the POL scenario always results in higher annual emissions than the other scenarios do. In contrast to the TECH scenario, in which the implementation of ambitious technical solutions takes relatively long to achieve, in the MOD and SUF scenarios, which both include sufficiency measures, GHG emissions are reduced quite quickly between 2023 and 2050. However, compared to those in the MOD and SUF scenarios, in the TECH scenario, lower GHG emissions are achieved from approximately 2037 to 2044; then, this scenario is surpassed by the SUF scenario. This dynamic shows that sufficiency measures can quickly reduce the short-term GHG emissions of the building stock, leaving time for the implementation of technical measures in the medium to long-term. Nonetheless, sufficiency measures also have long-term positive effects, as the SUF scenario shows.

The scenarios also represent different futures for key variables, such as the built area or the shares of heating systems in the building stock (provided in the Supporting Information). Compared with the POL scenario, the TECH scenario leads to little change in the number of new buildings (6% fewer due to fewer demolitions). Compared with the POL scenario, the MOD scenario leads the number of new buildings to decrease by more than half (60%), whereas in the SUF and AMB scenarios, almost no new buildings are built (the built-up area decreases by 93% compared with that in the POL scenario). Concerning energy carriers, in the POL, SUF, and MOD scenarios, there are still oil and gas boilers in 2050, accounting for 26, 11, and 10%, respectively, of the final heating consumption. In the TECH and AMB scenarios, fossil energy carriers are phased out by 2037, with a strong dependence on wood-based heating systems (54%), followed by district heating (24%) and heat pumps or other direct electric heating (23%).

### 3.2 Cumulative GHG Emissions and Remaining Carbon Budget

To compare the results with the remaining carbon budget, the cumulative GHG emissions of the scenarios (i.e., the sum of all emissions for all years) from 2023 to 2050 must be considered, as shown in Figure 4b. Although the POL scenario has the potential to reduce future GHG emissions by 66%, a cumulative 467 MtCO_2_eq will be emitted over this period, which is still 38% above the upper limit of the 2 °C carbon budget. Compared with the SSPs,^53^ this path seems to lead more directly toward a 3 °C future. This answers the first research question and shows that current policies are far from sufficient to meet the remaining carbon budget, which is a result shared in the literature.^54^

Four scenarios, each of which represents different societal choices, could enable conformity to the 2 °C carbon budget, which answers the second research question. In the SUF scenario, the average living space per person is reduced by 11% between 2023 and 2050, and 35% of vacant dwellings are utilized. By 2050, 90% of buildings set to be demolished are renovated and put to new use. Personal energy consumption is reduced by 20% by 2050. However, there is little pressure on industry because oil and gas, for example, will still be used for energy in 2050. In contrast, in the TECH scenario, the average living space continues to increase, growing by approximately 20% by 2050. However, fossil fuels are completely phased out in all energy systems between 2035 and 2040. Twenty nine percent of components are reused in 2050, and materials recycling is taken to extremes, e.g., with a 75% share of steel scrap in steel production. CCS is used on a massive scale and captures 90% of process emissions from cement production in 2050. The MOD scenario shows a moderate course in which the average living space increases by 7% by 2050, approximately 20% of vacant dwellings are used, and personal energy consumption is reduced by only 10%. Only 42% of process emissions from cement production are captured in 2050, and 10% of components are reused. The AMB scenario, which would require massive societal changes by all actors, combines the extreme values from both the SUF and the TECH scenarios and shows that, with our model, it is not possible to stay below 1.5 °C.

### 3.3 Influences of the Model Parameters and Assumptions

**Figure 5:**
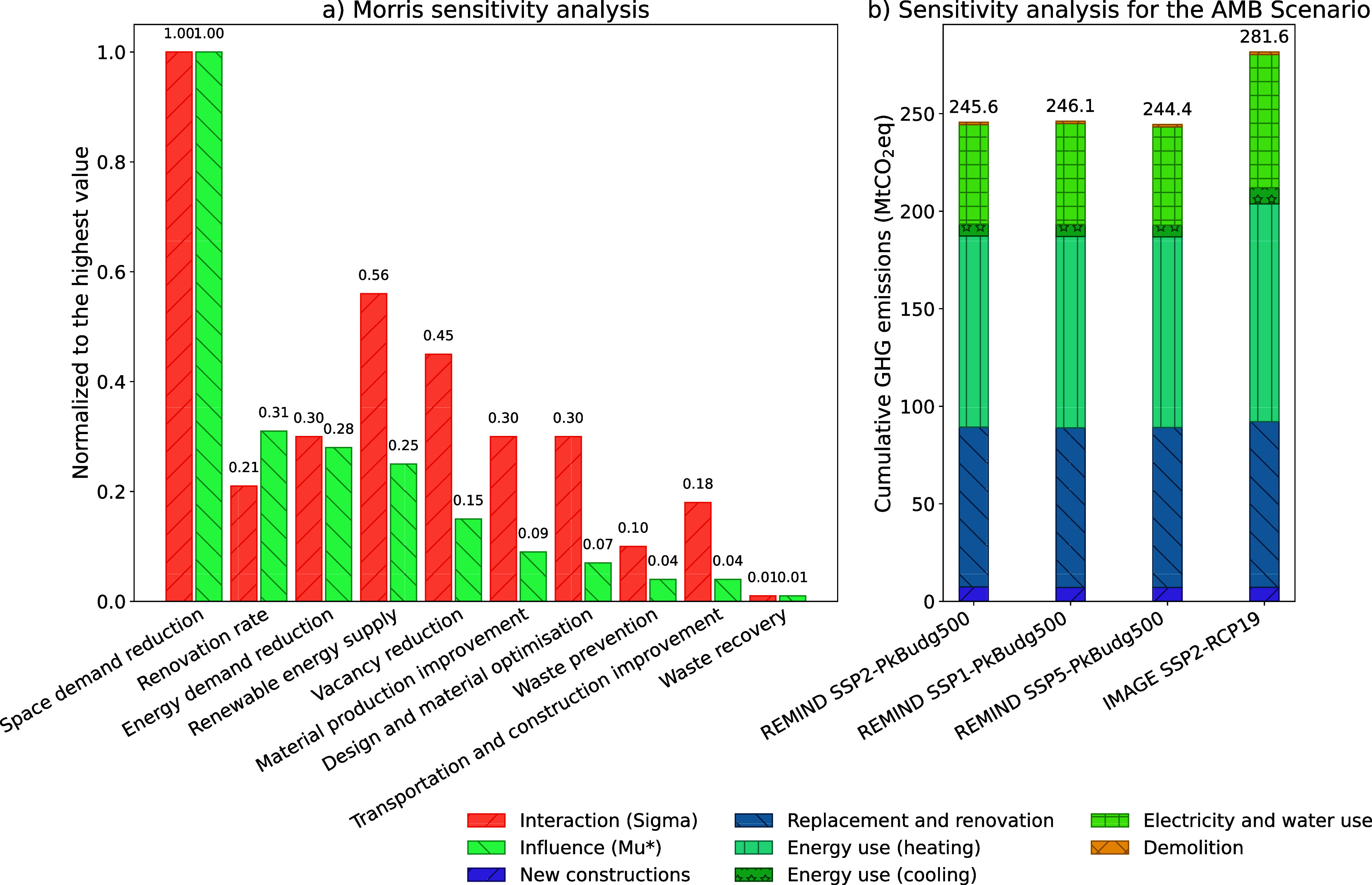
(a) Results of the Morris sensitivity analysis, shown as the mean elementary effect and standard deviation of the elementary effect (normalized to the highest value), and (b) results of the sensitivity analysis for the ambitious scenario, shown as cumulative GHG emissions for the different background LCA scenarios.

Figure 5a shows the ranking of the parameters according to the mean elementary effect from the Morris method (overall influence of the parameter on the output) and the standard deviation (level of interaction of the parameter). The five most influential parameters are space demand reduction, renovation rate, energy demand reduction, renewable energy supply, and vacancy reduction. Three of these parameters are sufficiency measures, which explains why the SUF scenario achieves such low GHG emissions. The values of renovation rate and renewable energy supply were expected, considering that Austria is a cold-climate country. However, it is interesting that space demand reduction is by far the most influential parameter, even exceeding the influence of the renovation rate, which may indicate that there are more interactions between the parameters than the one identified. These results could, however, be further validated using robust global sensitivity analysis methods (e.g., Sobol).

Figure 5b shows the difference in cumulative GHG emissions for the AMB scenario when the background LCA database is changed to a different SSP or IAM model. A less than 1% difference is observed between SSP2 and SSP1 or SSP5 within the REMIND model, which reveals the low sensitivity of the SSP narrative (as long as the target radiative forcing does not change). However, since SSPs differ in their assumptions on population evolution, adjusting the population in PULSE-AT scenarios could further refine the analysis and reveal greater differences. Changing the IAM model strongly influences the results, with a difference of almost 15% when the IMAGE model is used instead of REMIND. The largest differences come from energy consumption, especially electricity. This may stem from geographical differences between the IAM models or from differences in the allocation of emissions reductions among regions or sectors.

## 4 Discussion

### 4.1 Feasibility of the 2.0 °C Scenarios

A key finding of our study is that four possible trajectories could enable conformity to the 2.0 °C carbon budget, and one of them does not require sufficiency measures. This result differs from those of previous studies, which showed that achieving climate targets is possible only if 80% fewer new buildings are built and the operational energy demand is reduced by 80%.^54^ Other studies focusing on only energy-related emissions in buildings have noted that behavioral changes are necessary to sufficiently reduce GHG emissions from buildings.^55,56^ Although theoretically possible, we consider both the SUF and TECH scenarios to be unlikely due to the extreme measures required to achieve these GHG emission reductions. For example, we have received feedback from stakeholders of previous projects that a 75% share of steel scrap in steel production is far too high.^57^ These scenarios are also difficult to reconcile with actors who are economically dependent on the construction sector, as we may not build any new buildings at all under these scenarios. In contrast to previous studies, we show a different path, namely, the MOD scenario, in which all actors are quickly integrated, which leads to less extreme measures while enabling the achievement of the 2.0 °C carbon budget.

Now that we have shown a possible way forward, it needs to be translated into policies and implemented. Scholars have analyzed 1500 climate policy measures implemented in the last 25 years and identified the 63 most successful measures.^48^ They reported that popular instruments such as bans, building regulations, energy efficiency targets and subsidies are most efficient as a package of measures rather than as individual measures. In developed economies, prices and subsidies are the most complementary instruments. Promoting sufficiency is often perceived to be complex, especially when compared with technical emission reduction,^58^ but a variety of policy instruments have already shown promising results in reducing space demand, which is the most influential parameter of our model. Examples include raising awareness, rethinking communication, and designing effective financial motivations, such as combinations of different financial incentives that reward downsizing through flat swaps or minimum occupancy rates modeled on social housing in Zurich.^58^ Reducing vacancies is also among the top five contributors, and it has been shown that in France, a tax on vacant housing was responsible for a 13% decrease in vacancy rates between 1997 and 2001 (with most vacant dwellings becoming primary residences), which shows that this lever can be deployed rather rapidly.^59^ Finally, the MOD scenario’s emissions trajectory could be used to set limits on GHG emissions from new buildings.

### 4.2 Staying below 1.5 °C

Even in our most ambitious scenario, the 1.5 °C budget is not reached. This is plausible considering that we already have an average increase in the global temperature of 1.19 °C.^2^ A recent global publication shows that the most ambitious mitigation pathways manage only to limit maximum warming to 1.6 °C,^60^ which is consistent with our conclusion that reaching 1.5 °C is not possible without overshooting GHG emissions and capturing them between 2050 and 2100. Furthermore, they found that feasibility constraints, especially in the institutional dimension, significantly reduce this probability. However, active CO_2_ removal technologies are not included in our model, and their inclusion could change some of the results of the analysis. The REMIND SSP2-PkBudg500 scenario that we used for the background of the AMB scenario implies massive carbon dioxide removal starting even before 2050, which would also need to be translated to the context of building stock modeling to enable consistency and compliance with the 1.5 °C budget. Extending the time horizon to the year 2100 and include CO_2_ removals until 2100 could be explored in future studies. Generally, the remaining emissions in 2050 for the four scenarios that are below the 2 °C carbon budget range from 2.9 to 4.7 MtCO_2_eq. These emissions also need to be offset to reach net zero, and the choice of trajectory also influences the efforts that should be made to offset these emissions. Scaling up existing solutions in a very short time frame is often questioned and should be further investigated.^61^ Biogenic carbon storage due to the increased use of biobased materials is also not considered in this analysis. This can have a significant effect on GHG emission trajectories, although it implies land use change considerations.^62,63^ More research is needed to model the dynamics between biogenic carbon storage in buildings and the remaining carbon budget.

### 4.3 Carbon Budget Calculation

We compared our results to a carbon budget for the Austrian building stock that we calculated using the equal-per-capita allocation approach.^34^ In carbon budget calculations, there is rarely complete agreement on the allocation methods, and different approaches to the calculation can be considered legitimate. Fairness considerations of existing allocation principles, from the global scale to the national scale, are widely discussed in the literature, and no general agreement has emerged.^35,64−66^ For example, including responsibility for emissions since the Paris Agreement would limit the 2 °C carbon budget for the Austrian building stock to a maximum of 273 MtCO_2_eq, which would be reached only in the AMB scenario. In contrast, allocating the budget on the basis of the grandfathering approach, although widely considered unfair, would increase it to 698 MtCO_2_eq, which would be reached even in the POL scenario.^34^ The carbon budget should, therefore, be seen only as an indication of necessary GHG emission reductions, with significant uncertainty behind its calculation.

## Supporting Information Available

Parameters, scenarios, output, energy, and sensitivity (XLSX)
